# Neonicotinoid seed treatment on sugar beet in England: a qualitative analysis of the controversy, existing policy and viability of alternatives

**DOI:** 10.1007/s13412-023-00830-z

**Published:** 2023-04-15

**Authors:** Hannah Romanowski, Lauren Blake

**Affiliations:** School of Geographical Sciences, University of Bristol, Bristol, UK

**Keywords:** neonicotinoids, sugar beet, policy analysis, integrated pest management, pesticides, polarisation

## Abstract

In 2021, the United Kingdom Government granted the possibility of an emergency derogation for the use of the neonicotinoid seed treatment, thiamethoxam, on sugar beet in England. This was met with heavy criticism and controversy due to the body of evidence demonstrating toxicity of the insecticide to non-target species, particularly pollinators. However, many viewed this decision to be reasonable in this system, as sugar beet is a non-flowering crop, and derogations were only implemented if a set of conditions, including viral risk, were met. This research aims to understand the policy and the perspective of stakeholders in this debate, and identify key problems associated with thiamethoxam use on sugar beet. Semi-structured interviews combined with a modified policy analysis were used, incorporating framework analysis and comparative analysis. Political polarisation, whereby respondents felt that the debate had become anti-pesticide or pro-pesticide and lacked nuance, and the monopsony of British Sugar (a UK company that buys and processes sugar beet), were found to be the most prevalent issues currently impeding political progress and the enhancement of sustainable agriculture in this system. Virus forecasting was considered a successful strategy at the time of writing, although limitations to the model are also discussed. Non-chemical alternatives were found to be limited in this system due to the specificity of the pest system and the low threshold of virus yellows, while forecasting was considered to have the lowest net-environmental impact. Additional policy strategies to work alongside forecasting, such as public education and intergroup contact are also discussed. This study reflects a more general tug-of-war that often sets up a false dichotomy between food security and environmental sustainability. It highlights the importance of addressing the complexity of sustainable food production by opening up the discussion and taking a more nuanced and adaptive approach to policy.

## Introduction

Since the release of Rachel Carson’s acclaimed book *Silent Spring*, the use of pesticides has become a widely recognised environmental and health concern. Carson (1962) exposed the reality of previously celebrated chemicals, and initiated the ongoing dispute between the benefits and risks of pesticides. Since then, chemicals have come and gone from the market. The most well recognised example of this is dichlorodiphenyltrichloroethane (DDT), an insecticide first discovered during World War 2, now heavily associated with environmentally harmful effects (Turusov et al. 2002). The exposure of DDT as a highly toxic substance to non-target species and humans in *Silent Spring* eventually led to its ban in the United Kingdom (UK) in 1986, causing huge concerns for the agricultural sector (Buckley, 1986). It is this threat to growers’ livelihoods and food security that makes pesticide policy so convoluted. In an industry that relies on high yield outputs and intensive labour, pesticides provide effective, economical, reliable solutions to pest management.

Issues of food security are increasingly linked to pest management, as strategies that suppress yield damage by pests contribute to a reliable and efficient production of food. However, the unintended impacts of pesticides on natural pollination and other ecosystem services may ultimately be detrimental to food security (Brittain et al. 2010; Taylor, 2017). As the global population approaches 8 billion people, and pressure for high crop yields increases, it is important to understand this delicate balance, so that pest management is working for, not against, sustainable food production (Roser et al. 2021). As aptly summarised by Goulson (2013, p.985) it is about providing ‘the optimum balance between meeting the demands of food production and farming profitability in the short term, vs. the need to sustainably manage global biodiversity to ensure the long- term health of ecosystems (including farmland) upon which all life depends.’ The question then becomes, does that translate to a system with or without pesticides?

Neonicotinoids are a group of neuroactive synthetic insecticides, used to control a range of pest species (Bass and Field, 2018). They could be described as the modern-day DDT, as they too have brought about huge controversy regarding their environmental impact. Paradoxically, they were initially considered a safe alternative to DDT due to their systemic application, one that is absorbed by the plant and distributed throughout its tissue, as opposed to spraying. However, since their introduction in the early 90s, like DDT, an increasing body of evidence has been published highlighting their risk to non-target species, particularly pollinators (DEFRA, 2013; Godfray et al. 2014; Woodcock et al. 2016; Woodcock et al. 2017). In recent years this has led to great media attention, revealing the findings of research and leading to a fiercely debated narrative involving various stakeholders.

To the relief of many, in 2018, the outdoor use of all neonicotinoids compounds was banned across the European Union (EU) by the European Commission after considering the evidence presented in a report by the European Food Safety Authority (EFSA) (EFSA, 2018; European Commission, 2018). Since 2021 however, the UK government, no longer part of the EU, has authorised an emergency derogation for the use of the neonicotinoid compound thiamethoxam on sugar beet (GOV UK, 2023). This derogation was not limited to the UK, with a report finding that between 2019 and 2022, 57 derogations of thiamethoxam took place across the EU, in member states such as France, Austria, Denmark, Belgium, Cyprus, Greece, Hungary and Finland (PAN Europe, 2023). This was met with heavy criticism from environmental lobbyists, beekeepers, and international NGO’s, particularly as insect populations are known to be experiencing long-term declines in the UK (Bell et al. 2020; PAN UK, 2021). The decision was considered not precautionary enough and a neglect of the ‘body of evidence detailing the negative impact of neonicotinoids on not just bees and pollinators but also birds and other wildlife’ (PAN UK, 2021, p.1, Jensen et al. 2015). The emergency derogation however is limited by certain restrictions such as use only being permitted if predicted virus incidence level is beyond a threshold determined by Rothamsted YV (yellows virus) models, not allowing further use of thiamethoxam on the same field within 46 months of first use, reduced application and drilling rates of treated seeds, and herbicide use to treat weeds in treated fields (GOV UK, 2023). For example, the threshold was not exceeded in 2021 and so thiamethoxam was conclusively not authorised, however in 2022 the forecasted incidence of the virus well exceeded the threshold and so thiamethoxam was authorised for use on sugar beet in England (GOV UK, 2023; BBRO, 2022). As a biennial plant, sugar beet is also considered relatively safe, as it does not flower before harvest and is therefore not as attractive to pollinators. Sugar beet is also of particular economic concern, as aphid vectors of the virus, that thiamethoxam targets, are showing a high degree of resistance to pyrethroid pesticides, the alternative to neonicotinoids, and yield losses can be devastating (Bass et al. 2014).

This research project investigates the topics of controversy surrounding neonicotinoids, with a specific focus on the use of thiamethoxam on sugar beet in England. Qualitative research methods were used in the form of a policy analysis to gain detailed, constructive insights into the perspectives of stakeholders in this discussion, so that the policy problem and subsequent preferable policy options could be identified. Data was collected using semi-structured interviews with relevant respondents combined with review of policy documents. Alternative pest management strategies were evaluated using comparative analysis techniques. Triangulating the methods provides a unique perspective on this narrative, and is the first study to qualitatively review the use of virus forecasting in this system to reduce pesticide use.

Overall, the study aimed to identify key problems associated with thiamethoxam use on sugar beet, evaluate the use of virus forecasting, and investigate the viability of non-chemical alternatives to neonicotinoids. For coherence, this has been divided into three distinct research questions (RQ):

- RQ1: What are the main stakeholder perspectives on using thiamethoxam on sugar beet in England?
- RQ2:How successful has forecasting been in 2021 and 2022 at reducing the use of neonicotinoids whilst maintaining crop success?
- RQ3:What viable alternatives are there to the use of thiamethoxam seed treatment?

## Background

### Clarification of terms

In the context of this review, the following definitions of key terms will be used. Neonicotinoids are a class of neuroactive synthetic insecticide used to control a variety of crop pest species (Bass and Field, 2018). There are currently seven commercial compounds within the family of neonicotinoids, all of which are neuroactive by acting as an agonist on the nicotinic acetylcholine receptors of insect pests (nAChRs) (Buszewski et al. 2019), i.e. they overstimulate the nervous system of insects changing their normal behaviour and causing cell death (Simon-Delso et al. 2015). Thiamethoxam is the compound primarily used to treat sugar beet in the UK, and is a second-generation neonicotinoid first marketed by Cruiser® in 1998 (Maienfisch et al. 2001). Other countries that yield large amounts of sugar beet, such as France, Germany and Poland, when authorised also use thiamethoxam alongside other neonicotinoids to treat sugar beet (EFSA, 2021). To treat sugar beet, thiamethoxam is applied as a seed dressing, so is taken up by the plant tissue as it grows, and is distributed systemically across the entire plant.

There are minor variations in the interpretation of Integrated Pest Management (IPM), mainly around the specific strategies considered part of an IPM approach. For this study, IPM is defined using the European Commission’s IPM principles, that IPM means ‘careful consideration of all available plant protection methods and subsequent integration of appropriate measures that discourage the development of populations of harmful organisms and keeps the use of plant protection products and other forms of intervention to levels that are economically and ecologically justified and reduce or minimise the risk to human health and the environment (European Commission, 2023). IPM approaches also encourage a ‘prevention’ over ‘intervention’ approach, meaning that strategies focus on long-term prevention and control of pests as supposed to short-term interventions (I.e. chemical application) (Alston, 2011). This is a widely accepted definition, consistent with the Department for Environment, Food and Rural Affairs’ (DEFRA) definition in consideration of the UK focus of this study.

‘Sustainable’ is a contested term, and this is no exception when discussing sustainable agriculture. Whilst there are multiple attempts to define it, scholars and practitioners highlight the intertwined nature of social, economic and environmental sustainability within sustainable agriculture systems and more recently with a focus on regenerative farming and food systems (see for example Brodt et al. 2011; FAO, 2014; Duncan et al. 2020). The term ‘sustainable’ in relation to agricultural practices is defined here according to the four principles of sustainable agriculture of Trigo et al. (2021); (i) integrated management; which refers to a coordinated approach to management that considers agro-ecology principles for food production, e.g. nature-based practices, (ii) dynamic balance; which highlights a need for flexible yet stable farming through continuous assessment, evaluation and adaptation of practices, (iii) regenerative design; this refers to agricultural and economic practices within circular systems, within planetary boundaries and protecting and restoring ecosystems and (iv) social development, referring to those whose livelihoods depend on agriculture, and therefore by definition must also be considered within the sustainability framework. This definition was chosen because it identifies these principles from a recent meta-analysis of research and recommends its use in analysis.

Stakeholders are defined here as ‘actors who have an interest in the issue considered, who are affected by the issue, or who because of their position have or could have an active or passive influence on the decision-making and implementation processes’ (Varvasovszky and Brugha, 2000, p.341). In the context of this research, this can be interpreted as any individual or organisation that has an interest or is affected by the use of thiamethoxam on sugar beet. For example, the top five stakeholders here include British Sugar (sole processor of UK sugar beet), sugar beet growers, Syngenta (primary manufacturer of thiamethoxam), environmental organisations lobbying against the use of thiamethoxam (e.g The Wildlife Trust) and research institutes working with this system.

### Impact of neonicotinoids

The aim of neonicotinoids is to target a specific pest that diminishes crop yield, in the case of sugar beet, thiamethoxam is used to target the aphid vector *Myzus persicae* (Bass and Field, 2018). However, the reality is that it is difficult to isolate a single pest, and often there are impacts on non-target species and the environment (Botías et al. 2016; Goulson, 2013). Since their global introduction over 30 years ago, the environmental impact of neonicotinoids has become a concern, as an increasing body of evidence has highlighted multiple effects, particularly on pollinator species (Wood and Goulson, 2017). As the world’s most widely used and acclaimed class of insecticide, this has caused controversy, with many stakeholders suggesting that the evidence is not sufficient, and under and over-interpretation of scientific results has lead to confusion and mistrust in knowledge (Devine and Furlong, 2007; Godfray et al. 2014). The following evidence refers more generally to any of the seven commercial neonicotinoid compounds, unless otherwise stated to refer to thiamethoxam specifically.

#### Impact on pollinator species

While neonicotinoids were initially acclaimed for their relative specificity as a seed treatment, there is a large body of evidence showing extensive impacts on non-target insects. Since the nAChR receptor is highly specific to insect species, all neonicotinoid compounds have low toxicity to vertebrates, however non-target insect species remain at risk if exposed (Tomizawa and Casida, 2005; Moffatt et al. 2016). This has been of particular concern for pollinator species such as bees since traces of neonicotinoids have been found in the pollen and nectar of flowering crops and neighbouring wild plants (Bonmatin et al. 2003; Botías et al. 2015). Laboratory studies have been used to test the effects of neonicotinoid insecticides on bee species (Cresswell, 2011; Bryden et al. 2013; Main et al. 2018). Whilst there is little evidence to suggest that neonicotinoids directly impact mortality, these lab studies have found a variety of sub-lethal effects on numerous pollinator species, including reduced colony performance (Laycock et al. 2014), reduced queen production and colony size (Whitehorn et al. 2012), changes in foraging behaviour (Mommaerts et al. 2010) and reduction in feeding (Baron et al.*,* 2017; Laycock et al*.* 2012). It is important to note that regulation of insecticides is based on whether mortality of non-target organisms exceeds a level of concern based on measures of toxicity (e.g., dose that kills 50% of individuals relative to expected environmental concentration). However, the toxicity of neonicotinoids to bees through low doses in non-target flowering plants is difficult to test for, as long-term chronic tests on short-living insects is hard to control for, and are usually only a maximum of a few weeks long (Boily et al. 2013; Stanley and Raine. 2017; Thompson et al. 2019).

There are other limitations of laboratory studies that weaken the evidence in real-world conditions. For example, a 2014 review highlighted the effect of stress on the response of pollinator species to neonicotinoids, and criticised laboratory conditions for being either more or less stressful than field conditions (Godfray et al. 2014; Thomson, 2012). The same review touched on the methods used by laboratory experiments to introduce neonicotinoids to treatment groups, and warned that feeding a sugar solution to pollinators, as supposed to natural food collection, may affect insect species differently. Laboratory studies have also been criticised for using mostly honeybees (*Apis mellifera*) or bumble bees (*Bombus terristris*) as study species (Walters, 2013). In particular, solitary bee species, which are not buffered by colonies and therefore whose populations are likely to be more vulnerable to neonicotinoids, are studied less.

Considering these limitations, there have been various attempts at supporting laboratory studies with field-based experiments and observations. One of the largest field experiments was conducted by Woodcock et al. (2017), and assessed the effects of clothianidin and thiamethoxam on three bee species in Germany, Hungary and the UK. The results varied depending on species, country and neonicotinoid compound. In Hungary and the UK, neonicotinoid seed treatments had a negative effect on interannual reproductive potential and colony size, but in Germany, colony size was positively affected (Woodcock et al. 2017). This demonstrates how effects on pollinators are likely to be a product of multiple interacting factors, and that the results found in the laboratory experiments are challenging to replicate in realistic field conditions (Pilling et al. 2013; Chan et al. 2021). Overall, the evidence is considered insufficient to conclude that neonicotinoids are the *exclusive* cause of declines in pollinators (Laycock et al*.* 2012; Staveley et al*.* 2014). It is more likely that the chemicals are contributing to declines, amongst other important factors such as habitat loss and climate change (Bowler, 2021). Woodcock et al. (2017) for example suggested that large healthy hives can deal with exposure to neonicotinoids, whilst hives with a poor diet and an increased risk of disease are impacted by exposure.

#### Wider environmental and indirect impacts

Dave Goulson (2013) expanded on the evidence in a review that found neonicotinoids to be persistent in soils and detected in groundwater, streams, ponds and tidal creeks. This in turn raised concerns of exposure to other non-target species such as birds, mammals, and due to run off into water sources, aquatic species such as fish and crustaceans (Rodrigues et al. 2010; Lopez-Antia et al. 2013; Morrissey et al. 2015). A recent study also found that in response to neonicotinoid seed treatment, there were significant effects on the phyllosphere and soil bacterial communities, including declines in beneficial bacteria such as rhizobia (Parizadeh et al. 2021). Thiamethoxam has also been found to reduce microbial soil community diversity (Yu et al. 2020). Such impacts are particularly relevant here as they concern the agricultural industry in a way that potentially impacts long-term food productivity.

This also highlights the importance of considering indirect impacts of pesticides (Devine and Furlong, 2007). For example, another study found that imidacloprid was highly toxic to a natural enemy of the pest, the Colorado potato beetle, suggesting an antagonistic relationship between biocontrol approaches and chemical pest management (Lucas et al. 2004). This supports the view that IPM is not compatible with neonicotinoid use, as the inadvertent secondary impacts are considered detrimental to the integral concept and aim of IPM in the long-term, as well as them being prophylactically used, before the pest has invaded the crop (Tooker et al. 2017). More broadly, loss or change to one component of an ecosystem, in this example from neonicotinoid use (i.e. pollinators), can have cascading effects on entire ecosystems (Devine and Furlong, 2007).

### Thiamethoxam and sugar beet

As previously mentioned, thiamethoxam is a N-nitroguanidine neonicotinoid compound used as a seed dressing on sugar beet. In this system, it is mostly used to target the aphid species *Myzus persicae*, which is responsible for transmitting the yield-diminishing group of viruses known as virus yellows (Watson, 1946). There are three main types of virus yellows found in the UK; Beet Chlorosis Virus (BChV), Beet Mild Yellowing Virus (BMYV) and Beet Yellows Virus (BYV), all of which have devastating impacts on crop yield (Bayer, 2021, Dewar and Qi, 2021).

Based on evidence such as that presented in section Impact of neonicotinoids, thiamethoxam was first banned in the UK in 2013 by the European Commission. This was only enforced on flowering crops that were deemed a risk to pollinators (European Commission, 2013), and so thiamethoxam remained authorised for use on sugar beet in England until April 2018, when a report by EFSA prompted a full ban on all outdoor use across the European Union (EFSA, 2018; European Commission, 2018). These restrictions were lifted for thiamethoxam in England at the start of 2021, when the UK government authorised an emergency derogation (GOV UK, 2021). This decision heightened tensions and was heavily criticised, primarily by environmental non-governmental organisations and lobbyists, for not complying with pre-Brexit guarantees (PAN UK, 2021). The justification for meeting the three predetermined government requirements for emergency authorisation included the extent of damage caused by virus yellows, the lack of alternative means of protection and the use of virus forecasting with a threshold to ensure use was necessary (GOV UK, 2021). Regardless of this however, there was mistrust amongst lobbyists that this was warranted, and concerns remained regarding non-target flowering plants and insects. For example, whilst sugar beet itself is a biennial crop and does not flower before harvest, there is significant evidence that non-target flowering plants still take up neonicotinoids in following years, or by contamination of wild plants near to the crop (Woodcock et al. 2021; Botias et al. 2016; Sur and Stork, 2003). Just two months after the derogation announcement, the Rothamsted YV forecasts predicted that the incidence of virus yellows was below the threshold required for authorisation of use (9%) (BBRO, 2021b). Emergency use of thiamethoxam on sugar beet was therefore not authorised in England in 2021.

### Alternatives to neonicotinoids

One of the largest studies exploring alternatives to neonicotinoids was a case study that investigated the benefits and challenges of using IPM strategies in place of neonicotinoids in Italian maize production and Canadian forestry (Furlan and Kreutzweiser, 2015). Furlan and Kruetzweiser (2015) highlighted the potential success of IPM as an alternative to neonicotinoids in an agricultural setting, but concluded that it will take time to make this shift and will require investment in research and public extension. In a more recent and comprehensive review, Jactel et al. (2019) considered the efficacy, applicability, durability and practicability of alternatives in 152 cases of neonicotinoid use. Alternative chemical insecticides were the most commonly used alternative, however there were promising results in the use of microorganisms as biological controls, and semi-chemical physical controls such as using pheromones to disrupt of pest mating.

Alternatives to neonicotinoids in the sugar beet system have been less widely studied. This is perhaps a direct result of a delayed ban on non-flowering crops, or due to the efficacy of thiamethoxam in this pest system, both of which reduce incentive to research and develop alternatives. The focus has largely been on systems such as oilseed rape and maize, that were diminished after the 2013 ban on neonicotinoids (Scott and Bilsborrow, 2019). A study in 2017 however, did compare the use of thiamethoxam and alternative strategies on sugar beet, as a pre-emptive measure to an expected ban (Hauer et al. 2017). IPM alternatives, such as biological controls, were considered unavailable, while pest resistant varieties of sugar beet were more promising, yet still lacked research. In a more recent study, non-neonicotinoid treatments were tested against aphids on sugar beet, and while some other insecticides were effective, biopesticides (non-chemical pesticides derived from natural materials) were much less effective (Laurent et al. 2023). Considering this gap in knowledge, our study looked into whether this was a due to an absence of research, or whether alternatives to neonicotinoids are ineffective in this system.

### Research gaps and contributions

Despite neonicotinoid research being relatively extensive, particularly regarding their environmental impact, there are still gaps in our understanding. This is particularly prevalent in the sugar beet system, in which we appear to have a good insight into the pest system, and yet alternative methods to thiamethoxam are not well-researched. Using a policy analysis, this project aims to investigate whether viable alternatives - particularly within the realm of IPM - exist in this system, and if not, explore the challenges and limitations inhibiting their use. There is also a lack of research appropriate for policy makers that summarises and evaluates the perspective of stakeholders. This is essential in environmental conflicts, as stakeholders represent first-hand experience of the issue and can provide a comprehensive insight for decision makers, as well forming a necessary framework for effective politics. Qualitative research methods, in other words the analysis of non-numeric descriptive data to understand concepts, beliefs, experiences and behaviours, are particularly useful in this instance as they provide an in-depth and practical understanding of social interactions occurring within a political framework (Ritchie and Spencer, 1994). While specific interactions between pesticides and the environment continue to be researched, generating a broader, big picture perspective of the issue is important. This study is also one of the first to assess the use of virus forecasting in this system.

## Methodology

**Fig. 1:**
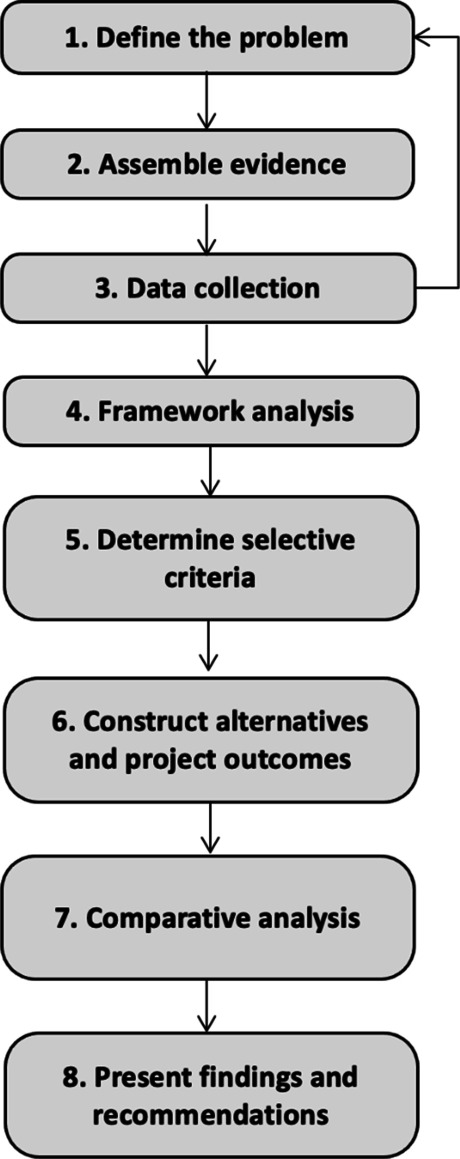
Policy analysis stages

A modified version of a policy analysis, based on the work of Bardach (2012) and Loomis and Helford (2003) and outlined in Fig. 1, was deemed most appropriate to answer the research questions. Qualitative methods in particular were thought to be more conducive to answering questions about experience and perspective (Hammarberg et al. 2016). Within the policy analysis methodology, data was analysed using thematic framework analysis of interview transcripts and documents, followed by a comparative analysis of alternative strategies. Weimer and Vining (2017, p.30) define policy analysis as ‘client-orientated advice relevant to public decisions and informed by social values’. More specifically, environmental policy analysis identifies environmental and social impacts of a current policy, and uses information to evaluate and present improvements for public decisions. This is particularly useful for this narrative, as neonicotinoid policy has developed a controversial status and regular modifications in policy suggests uncertainty in decision making. Adjustments to a traditional policy analysis followed the aims and trajectory of data collection in an iterative process. For example, the initial definition of the problem was redefined following data collection, and so steps 1 and 2 were repeated (Fig. 1).

### Data collection

Data was predominantly sourced from semi-structured interviews with nine respondents: one consultant ecologist, three research entomologists specialising in insect molecular biology, quantitative ecology and IPM, an NGO policy officer, three arable farmers (two of which grow sugar beet) and an agronomist (full anonymised details in Appendix 1). All interviews took place in July and August 2021. Sampling was purposive and participants were chosen based on their connection with thiamethoxam use on sugar beet and approached via email. Interviews took place in July and August 2021 and were conducted online due to Covid-19 restrictions in place at the time. Interviews were semi-structured to allow flexibility in the discussion, whilst retaining some control of the topics covered (Bryman, 2012a). Questions fell into four sections: (i) introductory questions on neonicotinoids and interviewee background, (ii) questions around the source of the controversy and ‘the problem’, (iii) alternatives to neonicotinoids, and (iv) integrated pest management (see Appendix 2 for interview guide template). Relevant policy documents from a range of government and non-governmental groups, as listed in Table 1, were also analysed alongside the interview transcripts to triangulate data against the literature and policy objectives for a comprehensive policy analysis. The policy documents were purposively selected based on their contribution to current policy development (within a recent timeframe) and their prominence in the grey literature.

**Table 1:** List of policy documents used in analysis

	Publication date	Title of document	Organisation(s)
1	Mar 2013	An assessment of key evidence about Neonicotinoids and bees	DEFRA
2	Jul 2017	Bees and neonicotinoids	House of Commons
3	Feb 2018	Farming Sugar Beet without Neonicotinoids	Friends of the Earth Pesticide Action Network UK Buglife
4	Nov 2018	Cutting pesticide use and promoting integrated pest management in UK agriculture – a farmer’s perspective	Friends of the Earth
5	Dec 2018	National pollinator strategy: implementation Plan, 2018-2021	DEFRA
6	Jan 2021	Organisations unite against neonicotinoid decisions	Pesticide Action Network UK
7	Jan 2021	Neonicotinoid product as seed treatment for sugar beet: emergency authorisation application	DEFRA GOV UK
8	May 2021	Statement on the decision to issue – with strict conditions – emergency authorisation to use a product containing a neonicotinoid to treat sugar beet seed in 2021	GOV UK

### Data analysis

Data was analysed using two different methods at two stages of the policy analysis; thematic framework analysis and comparative analysis. Thematic framework analysis analyses qualitative data by searching for repeated patterns that then aid the description and interpretation of the data (Kiger and Varpio, 2020), whilst comparative analysis refers to the comparison of policies using qualitative data to find similarities and differences. Both were undertaken on interview transcripts and the policy documents.

**Fig. 2:**
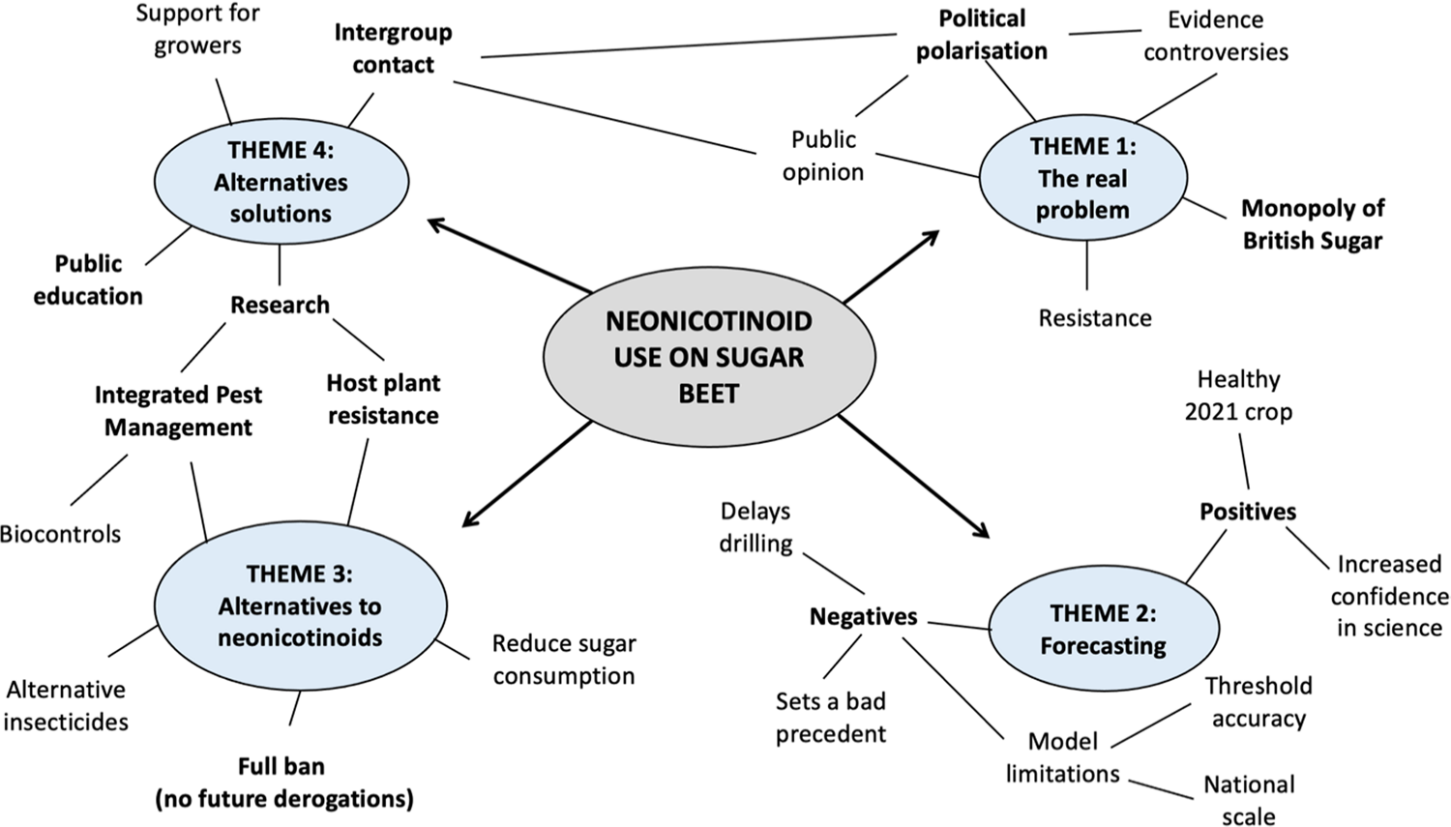
Schematic diagram of themes identified through framework analysis

Framework analysis was chosen as the most appropriate method of thematic analysis (drawing out themes from multiple sources of data), and involves five key stages; familiarisation, identifying a thematic framework, coding, charting and interpretation (Ritchie and Spencer, 1994; Srivastava and Thompson, 2009). Themes were initially recognised by reading transcripts for repetition, metaphors and analogies, transitions in conversation and unfamiliar vocabulary (Ryan and Bernard, 2003), with the same approach to policy documents. Each document was read at least three times; once for familiarisation, again for coding, and a final time to chart the data into a thematic framework. Coding was completed for both interviews and policy documents as recommended by Bryman (2012b). A combined inductive and deductive approach was taken to coding, bearing in mind the research questions around forecasting and alternatives, and themes identified from the interviews being considered against the policy documents, but with an open approach so that new themes and key findings outside of the emerging framework were not overlooked. The first few interview transcripts were analysed initially, producing the preliminary codes and themes, then the two datasets were analysed simultaneously, with both analyses feeding into each other to generate the thematic framework. Figure 2 shows a schematic diagram of the generated thematic framework.

Comparative analysis was used latterly to project outcomes of alternative policy strategies. This involved assembling an outcomes matrix (Table 2 and section 4.3), as suggested by Bardach (2012). Selective criteria were determined based on (i) the problem defined in stage 1, (ii) Bardach’s (2012) recommendations regarding commonly used criteria and (iii) themes from the framework analysis. Two categories of policy alternative were comparatively analysed; alternative pest management strategies and policies in addition to current policy. Four of the six criteria were the same for both categories, however some criteria was appropriate for one and not the other. A score from 0-3 measured criteria satisfaction, with each criteria weighted the same.

**Table 2:** Outcomes matrix of results from a comparative analysis of policy options

POLICY SCENARIO		CRITERIA								
		Minimise environmental impacts	Minimise risk to growers’ livelihoods	Maximise knowledge exchange	Minimise political polarisation	Political feasibility	Practical feasibility	Equity of stakeholder satisfaction	Longevity	Enhance understanding of biological system
ALTERNATIVE POLICY	Forecasting	2	2			3	2	2	2	
	Integrated Pest Management	3	1			1	0	0	1	
	Host plant resistance	3	3			0	0	3	1	
	Full ban	1	1			2	3	1	1	
IN CONJUNCTION WITH CURRENT POLICY	Public engagement			3	2	3	2	2		1
	Intergroup contact			3	3	3	3	3		0
	Farmer driven research			2	1	2	2	2		3
	Host plant resistance research			0	1	2	1	2		2
	System specific IPM research			0	1	2	2	2		3

Level of criteria satisfaction is scored between 0 and 3 whereby: 0 is negligible, 1 is low, 2 is medium, and 3 is high

### Ethical considerations

The four main ethical principles of social research categorised by Diener and Crandall (1978) - harm to participants, informed consent, invasion of privacy and deception - were considered in detail before research began. Each respondent received a participant information sheet with details of the purpose of the research and what the process would involve, with time given to consider and ask questions, followed by a consent form that was compulsory to complete before the interview. Anonymity of respondents was maintained throughout. Ethical approval was given in June 2021 by the School of Geographical Sciences Research Ethics Committee, University of Bristol, reference RE-B-ROMANOWSKI-20210614.

### Reflections

The main limitation of this study is the sample size – nine respondents could be considered too few. However, the interviewees represent key pertinent stakeholders with a range of perspectives and experiences relating to this topic. There was also a small degree of sampling bias in the form of self-selection (i.e. the non-random selection of participants due to their relevance, plus their interest and willingness to participate) and non-response. The latter was more prevalent here, as a lack of response from sugar beet industry organisations and Syngenta, the primary producer of thiamethoxam, meant that this perspective was not represented. This was mitigated by further supplementing interview data with in-depth analysis of policy documents and open-access statements made by British Sugar and Syngenta on this issue.

COVID-19 meant that interviews all took place online, often being the first time respondent and interviewer met, which limited the rapport built between respondent and interviewer that can be valuable to the interview process. WIFI connectivity issues also potentially resulted in loss of informative data due to inaudibility of the recording. The triangulation of data sources played a role in mitigating many of these limitations.

## Findings and discussion

Following the stages of the policy analysis format, this section begins with an evaluation of the problem, followed by a review of current policy, then a discussion of alternative pest management strategies, concluding with a discussion of policy alternatives and future recommendations.

Four main topics were identified from the analysis of the 9 stakeholder interviews and 8 policy documents (Table 1): the real problem, forecasting, alternative pest strategies and alternative policy solutions, each with subsequent themes (Fig. 2).

### Defining the problem

Uncertainty around the scientific evidence, as discussed in section 2.2, was expected to be the biggest concern amongst respondents. This however was not the case, and it was increasingly clear that concerns were more multifaceted and systemic than anticipated. This section focusses on two key problems regularly mentioned by respondents, however there were additional subthemes, such as public opinion, resistance to neonicotinoids and evidence controversies (Fig. 2). These did not fall within the scope of this project as they are either well researched or the data was too ambiguous to reliably draw conclusions.

#### Political polarisation

A recurrent problem raised by respondents was that the discussion on neonicotinoids has become polarised. Respondent 1, an insect molecular biologist with a background in neonicotinoids and crop protection, said “*Actually, I think the worst thing we did when there was the height of the issues around neonics was to polarise it. […] But when you start very frantic debates, especially when they’re political, they get polarised. That really doesn't help, because science isn't like that, it's not yes or no, is it?”*. The growers and agronomists interviewed also felt that the discussion had become polarised, and felt targeted negatively by the public on the issue. The NGO official expressed firm views against neonicotinoids under all circumstances.

Respondent 4, an ecologist with research experience in the thiamethoxam system, highlighted how the problem is not ‘black and white’:

*“You’ve either got to be anti-pesticide or pro-pesticide […] but I’ve worked with both sides and you know it's not a straight cut, it's not easy […] I guess it is just summarising to say that is not a black and white issue. It's not a yes or no answer […] but you know people need to listen to both sides and find a middle ground”*

Political polarisation in this context lacks research and understanding, however in other fields, such as climate change, it has been found to impede political progress (Lucas and Warman, 2018). Here, it is likely the conflicting stakes and priorities of opposing partisans that facilitates polarisation. The neonicotinoid debate, and the wider international discussion on pesticides, is generally perceived as a dichotomy, fuelled by media attention and the undermining of science (Lehrer and Sneegas, 2018; Orford, 2022). Cooperation is then neglected and misunderstandings persist, reinforcing the conflict. For example, one respondent here said that environmental lobbyists often misunderstand the environmental impact of a full ban in this system, which could for example lead to the outsourcing of potentially less sustainable produce.

In a broader review of neonicotinoids, Walters (2016) suggests a solution to the problem of polarisation: “Broadening of the debate to consider the complimentary objectives of bee conservation and sustainable crop production would therefore enable advances in both fields to be more readily used to hasten consensus on the way forward, surely preferable to our current polarised debate that reduces the prospect of such consensus being achieved” (p.381). As he infers, a more open discussion is required, which may be facilitated by strategies such as intergroup contact and the adoption of a more balanced approach that considers a wider range of perspectives.

#### Monopsony of British Sugar

The organisation of the sugar beet industry in England was also raised as a concern by multiple respondents. For clarification, local growers of sugar beet in England supply the monopsony (sole) buyer, British Sugar (BS), which driven by the market processes all the sugar beet in the UK. Respondent 4 described the contractual system:

*“Yeah so, […] sugar beet crops are controlled by British Sugar. So the farmer at the beginning of the season will know how much sugar beet they have to grow. And the contract will say you need to provide us with X tonnage of sugar beet, and if you don't provide that much you get fines for example. […] And they kind of have the monopoly, because it is just British Sugar”*

This arrangement encourages the use of neonicotinoids for two reasons. Firstly, pressure is put on growers to fulfil contracts based on yield, meaning that risks of using alternative pest management strategies are intensified. Respondent 5 said “I *can see why there’s so much pressure on them to use something like neonicotinoids […] It’s like if someone turned round to you and said “Would you be willing to take the risk to not get paid for an entire year? You have to do the work but you might not get paid at the end of it””.* Secondly, in years when neonicotinoids are authorised, the decision to treat the seed is made by BS not the growers (BS, 2018). This removes choice by growers to use alternatives and creates a barrier for new research. Respondent 9, an arable farmer that has historically grown sugar beet, explains:

*“British Sugar control[s] the seed supply tightly. Every variety has to go through British Sugar, and only British Sugar allow it onto the list that the farmer can select from […] So until British Sugar say we want a variety that is tolerant to yellows, there is no impetus for those companies to spend money developing it […] At one time you couldn't order seed that wasn't treated with neonicotinoid”*

Pressure to implement certain practises is not exclusive to the sugar beet system, and research suggests engagement not just with farmers but with the institutions that drive farm management decisions is important (Baur, 2020). Alternatives to this industry system are however politically challenging as the government was involved in authorising Associated British Foods’s purchase of BS to facilitate this monopsony, and BS has a history of encouraging its persistence (Raworth, 2002). Strategies to ease the symptoms of the problem, such as choice for growers and funding by BS for research, would be beneficial here.

### Forecasting

The process and results of 2021’s aphid forecast were explained by respondent 2, who works closely with the forecasts. They summarised that “*If you take the January/February temperature, mean temperature, and you forecast the first flight of the aphid, you can get very close to the estimate of what is actually observed in a suction trap”*. This estimate is then used to predict incidence of virus yellows and the resultant threat of the pest. In 2021, the forecast predicted that 8.37% of sugar beet in England would be infected, and since the 2021 threshold was 9%, the derogation was not authorised.

#### Positives of forecasting

The most recognisable positive of forecasting has been the direct impact on plant health. Respondent 7, a sugar beet grower that was hit badly by virus yellows in 2020 said “*In fairness the sugar beet’s looking pretty well. We’ve got two fields that are certainly looking as good as they have done for a few years. […] and we’ve got good cover of beet and it’s looking quite healthy at the moment’.* Respondent 8, another sugar beet grower, said ‘*Yeah, it’s worked I think. The crop has got to sort of a critical stage, […] So obviously the predictions on the modelling was correct and the virus is not going to be a major factor this year”.*

Although the crop is not harvested until autumn, growers use the 12 leaf stage as an indicator of mature plant resistance. A 2021 review by BBRO stated that ‘crops have made great progress over the last week with many now at the 10-12 leaf stage’ (BBRO, 2021a, p.1), indicating severe loss to yield by the virus would be unlikely in 2021. This was confirmed after harvest, when it was confirmed that sugar beet yield was 26% higher in 2021 compared to 2020 (DEFRA, 2022). This success is not only important for growers but also for research organisations that rely on public and stakeholder confidence to facilitate and fund further research. Respondent 1, who is familiar with these challenges, commented that ‘at least [forecasting] meant they were using the science’.

Evidence of high yields in the absence of neonicotinoid treatment may however facilitate a greater push from environmentalists for a full ban. The increased confidence in the science may also encourage industry to engage more with other alternative methods that would otherwise not have seemed worth the risk. Alternatively, forecasting may be a middle-ground necessary to moderate the polarisation of the argument, and encourage a more compromised approach, since 2021 has been a ‘success’ for both the environmental sector and the sugar beet industry.

#### Limitations of forecasting

Respondents also raised concerns regarding forecasting. Respondent 3, described how “*[Emergency derogation] is often abused. Certainly if you look at other EU Member States, derogations are basically rolling year on year. […] It also sets a very nasty precedent because they’ve got three years potentially approved for emergency derogations [in the UK]”.*

Weaknesses of the model were also highlighted. Firstly, since the model uses first flight of aphids to predict virus intensity, the final decision for 2021 not made until March of that year. Since seed drilling usually occurs at this time there is a risk that drilling will be pushed back, delaying the onset of mature plant resistance. Respondent 7 highlighted this:

*“The only slight concerns I’ve got with it is that by the time they’ve calculated the thresholds and then decided whether they need to dress the seed or not, it’s pushing our drilling back a little bit. The seed wasn’t on farm this year until sort of the end of March start of April. Sugar beet drilling time tends to be between the sort of 15* ^*th*^ *of March and 15*^*th*^ *of April traditionally. So if you’re in a year where you can get on early, I can see that that being a slight drawback to it”*

Secondly, although the science behind forecasting in this system has been studied for over 30 years, 2021 was the first year that it was been linked to government decisions and a threshold been set (Harrington et al. 1989). It is therefore difficult to establish whether the threshold is appropriate. Respondent 2 expressed this concern: “*We’re going to get it wrong sometimes. Particularly now we’re on the margin, you know. So 8.37 is not far from 9%, uhm, and what if we’re 9.1%, what do we do then?”.*

Respondent 7 shared this concern:

*“You know the problem is whether the threshold is set in the right place or not, and you don’t know that until you’ve had either a problem or a year where it’s been borderline and we’ve been allowed to use the neonics. And you know this year it worked okay, but you know is the threshold right or has it worked okay just because the conditions weren’t right for the aphids?”*

In the year after this research however (2022), the threshold was increased from 9% to 19%, showing a flexibility in setting the threshold, based on the experience of previous years (BBRO, 2022). Regardless, the forecasted incidence overwhelmingly exceeded the threshold in 2022, and so an emergency derogation of Cruiser SB was authorised (BBRO, 2022). As data was only collected in summer 2021, this study cannot conclude any further on the outcomes of the 2021 derogation.

Finally, the model uses suction trap data that determines a single national forecast. Virus incidence however is not spatially homogenous, and is likely to vary depending on region, field size, adjacent crops etc. Respondent 2 mentioned that information is available to enhance forecasts at a spatial scale, however various challenges need to be overcome before it can be applied here. The Rothamsted forecasting is also only used for sugar beet in the UK, which is also localised to the east England, and so is not yet applicable to other crop systems and countries.

### Comparative analysis of alternative pest management strategies

Comparisons between alternative pest management strategies to thiamethoxam use, and additional strategies to current policy are presented as a score matrix in Table 2, as recommended by Bardach (2012) for comparison and reference. Maximising knowledge exchange, minimising political polarisation and enhancing our understanding of the biological system were not used as criteria in the comparison of current policy. Minimising environmental impact, minimising risk to livelihood of growers and longevity were not used as criteria in the comparison of additional strategies in conjunction with current policy, however these criteria were considered for current policy (forecasting) in the first row of the matrix. A high level of criteria satisfaction (score of 3) was given to policies that were regularly suggested or mentioned as important in interviews or policy documents. A low level of criteria satisfaction (score of 0) was given to policies that were not mentioned or cannot currently satisfy this criterion at all. For example, host plant resistance is currently unavailable in sugar beet and therefore scored 0 for feasibility (Table 2), whilst forecasting, by the logic that it is being successfully implemented at present, scored highly for political feasibility.

#### Integrated Pest Management

Examples of IPM mentioned in interviews and document analysis included biocontrols (e.g introduction of predators), chemical ecology (e.g using pheromones to deter aphids), push-pull mechanisms (using other crops/plants close to main crop to ‘push’ pests to alternate areas and ‘pull’ them away from main crop), improved plant hygiene, reduced ploughing, rotations and intercropping. These examples were always discussed with positive implications, however not in the sugar beet and *Myzus persicae* pest system. For example, respondent 9 described their success in eliminating neonicotinoids from oilseed rape by ‘farming more sympathetically to insects’ and increasing rotations to reduce soil borne disease and resistance. Similarly, biocontrols were identified by two respondents as the most auspicious strategy for this system, however field trials tend to have studied either biocontrols on sugar beet crops or against the pest, never the two together (Shalaby and El-Nady, 2008; Galletti et al*.* 2008; Mohammed and Hatcher, 2017).

This study identified three reasons IPM fails in this system; (i) a low threshold for the virus, (ii) specificity of the system and (iii) lack of incentive for farmers. Respondent 5, an agronomist with experience in this system, said that “*[IPM] will only protect us to a certain extent […] and if they did arrive, no amount of cultural control is going to protect us”.* In terms of the system specificity, respondent 2 described how IPM strategies can even work against growers in this system:

*“And even this green agriculture that’s going forward, which I am a great advocate of, in the Myzus system it is a disaster. […] Basically the thinking is if you enrich the habitat with wildflowers, what you tend to be doing is introducing reservoirs for the virus. […] So in other systems it works really well, but in the Myzus instance it works against them […] it can also work against you if you don’t understand the system”*

Growers would therefore be taking a risk by using IPM, and as mentioned previously, even if growers are open to using IPM as an alternative to neonicotinoids, pressure and control from BS make this difficult. Despite pressure, farmers are often burdened with shouldering the, particularly financial, risk of making changes towards more sustainable practices, especially against a wider system that doesn’t necessarily reward this (Rodriguez et al*.* 2009; Coyne et al. 2019). This risk was reflected in the comparative analysis score, and beyond maximising environmental protection, IPM satisfied few of the criteria (Table 2). This included political feasibility, as IPM strategies would be challenging to implement in this system due to the lack of evidence that alternatives to neonicotinoids provide the optimal resolution between agronomic and ecological objectives. Also, as an example, the EU’s Sustainable Use Directive made IPM obligatory across all EU member states in 2009, however its uptake has been limited due to physical barriers and a lack of a clear definition of IPM (European Commission, 2009; Doonan, 2017). Since the UK is no longer part of the EU, this is an opportunity for the UK government to set out a clear action plan for IPM, highlighting clear definition and targets. In seeking to reduce pesticide (and nitrogen fertiliser) use generally in arable farming, Lechenet et al. (2014) showed that integrated approaches can bring a range of benefits without significant trade-offs (e.g. workload, yields, profitability).

#### Host plant resistance

Host Plant Resistance (HPR) is the development of a sugar beet plant that is either bred or genetically edited to be resistant to virus yellows. In general, respondents felt HPR was an ideal solution as it eliminates the need for pest control. It is also important to acknowledge here that no respondents were affiliated with Non-Governmental Organisation’s that explicitly oppose genetically modified organism (GMO) crops.

There is currently no variety of sugar beet that is fully resistant to all strains of the virus, and although ongoing breeding programmes exist, the recurring view was that it is not easy and takes time to achieve. Respondent 2 described this in more detail:

*“So in some systems, they’ve got a really rich genetic pool from which to dip into. But in sugar beet you haven’t […] basically complete plant resistance is difficult to achieve in sugar beet because of the limited genetic diversity. Those wild-type traits that you might import are not suitable for elite breeding programmes. […] I don’t know how much you know about this system, but you have 3 viruses [and] you’ve got 3 aphid vectors, and you’ve got multiple virus reservoirs. And so it’s unlikely, with such low genetic diversity, that a plant can truly be resistant to all 3 types”*

Maruscha KWS is a new variety that has partial tolerance to BMYV (May and Bowen*,* 2021). Although this is progress, this reiterates respondent 2’s point, that until a variety is developed that can reliably resist all three, this is not an effective solution. Derogations may be seen as a ‘stop-gap’ until a resistant variety is available. Longevity of HPR is another concern, due to the risk of new strains of the virus making new HPR varieties redundant.

#### Full ban of thiamethoxam

In January 2021, Pesticide Action Network UK wrote to the UK Government asking it to reconsider the decision to authorise an emergency derogation on the use of thiamethoxam on sugar beet due to ‘the body of evidence detailing the negative impact of neonicotinoids’ (PAN UK, 2021, p.1). The letter was signed by over 30 organisations including the World Wildlife Fund, The Wildlife Trust, Friends of the Earth (FOE) and the Royal Society for the Protection of Birds. The positives of this alternative are clear and represent the ‘precautionary principle’ approach to the environmental impacts of thiamethoxam (Patterson and McLean, 2019). Respondent 3 also highlighted how a ban could drive the development of IPM strategies, which was reinforced by a FOE document that claimed that ‘The prospect of the extension to the ban [in 2018] has already prompted industry led research into alternative ways to protect the sugar beet crop. These are focussed on proving forecasting and monitoring of aphids’ (FOE, 2018, p.11). Implementation, and the apparent success of forecasting, may therefore have been fuelled by a need for alternatives after the 2018 ban.

On the other hand, findings suggested that a ban of thiamethoxam, so the retraction of future derogations, may have a worse net environmental impact. Firstly, foliar sprays remain authorised and so are likely to be used more in the absence of thiamethoxam seed treatment*.* Foliar sprays are less targeted and contaminate a higher portion of the environment. Also, due to the current limitations of IPM strategies and HPR, when incidence of the virus is high, yield is unlikely to be sufficient to meet UK demand in the event of a ban. At present, approximately 50% of UK sugar is supplied by domestic sugar beet, 25% is sourced from European imports of sugar beet, and the remaining 25% is sourced from imported sugar cane (AB sugar, 2022). If the UK cannot meet demand, the proportion of imported sugar cane is likely to increase. This will not only waste resources (i.e land, seeds, water), but will incur a carbon footprint and may also involve the use of less restricted pesticides and less sustainable farming practices.

These consequences were highlighted by multiple respondents, including respondent 1 who said “*Shouldn't we be encouraging sugar beet to get sugar in this country rather than importing it from places that grow sugar cane? […] You’ve got to be really careful about the unintended consequences of these things’.* Respondent 2 similarly raised concerns of displacing the issue overseas, stating *‘I think that if we were successful at removing neonicotinoids from the whole of the British system, all that would do would export to another country that has less stringent controls […] and so all we would be doing would be displacing the problem”.*

These unintended consequences, or pesticide externalities, must be considered in net environmental cost calculations. This raises the question of how we quantify net environmental impact in a complex system with different metrics of impact. The pesticide environmental accounting (PEA) tool has been used for estimating costs of pesticides for policy and comparing pesticides to alternatives such as GM (Leach and Mumford, 2011). This type of tool would be beneficial here to quantitatively compare the environmental costs and benefits of these alternatives.

### Alternative solutions raised and recommendations

Since this study did not confirm a viable alternative to the use of thiamethoxam on sugar beet, options to be used alongside forecasting were also considered in the comparative analysis (Table 2).

#### Intergroup contact

Intergroup contact refers to the theory of creating contact between multiple stakeholders under appropriate conditions to reduce prejudice and alleviate tensions (Pettigrew and Tropp, 2006; Christ and Kauff, 2019). The intergroup contact hypothesis was first suggested by Allport in 1954, and is deemed most effective under four conditions: equal status, cooperation, common goals and support by authorities (Everett, 2013). Whilst this usually refers to face-to-face contact, which may be difficult in this context, recent research has found indirect method of intergroup contact to also be effective (Wright et al. 1997). Vicarious contact, or the observation of contact between different groups, is even thought to be more sustainable and able to reach more people than direct contact. Based on the comparative analysis, it is recommended that contact between stakeholders of this debate be encouraged and demonstrated to the public. Polarisation of environmental protection and food security is a false dichotomy, that in reality is far more complex and convoluted. Achieving a balance between meeting the demands of sugar production and farming profitability in the short term, whilst sustainably managing biodiversity and ensuring the long-term health of ecosystems, is a more realistic representation of the shared goal of stakeholders (Goulson, 2013). Intergroup contact will reinforce this and hopefully begin to shed light on the false and unnecessary dichotomy that is felt in this system. From a more pessimistic perspective, the polarisation of this debate may be too well established, however lessons can be learnt from this and cases of such extreme political polarisation may be prevented by earlier interventions such as intergroup contact.

#### Public education

Public education is used as an umbrella term here for recommendations aimed at enhancing public understanding on different aspects of this narrative. Firstly, findings showed net environmental impacts were often overlooked, particularly by those advocating a full ban. This is particularly concerning when considering that the externalities of a failed sugar beet crop are potentially more spatially and ecologically widespread than the impacts of thiamethoxam on pollinator species. Findings of this study also highlighted the complexity of Myzus-sugar beet system. Developing a clearer narrative on the challenges of IPM, the impacts of virus yellows on crops, and the way that BS controls the industry will encourage knowledge exchange that ultimately facilitates a reduction in political polarisation.

It is also important here to also acknowledge the role of the media and press in polarised debates such as this. Studies have found that social media can spread misinformation and misrepresent the nuance of an issue, that then results in further polarisation (Kubin and von Sikorski, 2021; Wilson et al. 2020). Since public opinion has a weight on policy decisions, it is important that the public understand these unintended consequences and are fully informed (Burstein, 2003). In particular, and in a wider context of polarised debates, a greater awareness of the trade-offs between policies and the danger of oversimplification is significant. This could happen through for example public awareness campaigns or direct training and support.

#### Research

These findings support the work of previous research that calls for a comprehensive approach to regulation and highlights the need for high quality data and strong scientific support (Hall et al. 2012; Bruce et al. 2022). Here, farmer driven research is essential to enhance our understanding of this system, as traditional research has been found to limit action through access issues and comprehensibility (Pressland, 2018). In the sugar beet system, bottom-up research would be beneficial in developing our understand of thiamethoxam in the environment and would give some control back to the growers. Funding and responsibility should therefore be distributed to farmers in exchange for their experience and land-use.

Current policy, in which there are years thiamethoxam is authorised and years it is not, also presents an opportunity for large scale field studies on ecological impacts. Most studies apply ‘field-realistic’ doses of thiamethoxam in a lab or experimental field studies, however this form of observational research at field level is limited (Godfray et al. 2014). A recent study in Germany observed 94 sugar beet sites in time periods after thiamethoxam seed treatment, and found that residues in soil, guttation fluid, pollen and nectar, were below sublethal doses to pollinators (Thompson et al. 2021). Studies like this could be conducted across sites in England, observing residues in a similar way, as well as the ecological implications.

## Conclusions

### Key findings

In seeking to understand the main stakeholder perspectives on using thiamethoxam on sugar beet in England (RQ1), as expected, respondent perspectives depended on their position in the discussion. Researchers and growers generally felt that when used responsibly and in the correct circumstances, thiamethoxam is a valuable tool. However, a policy officer campaigning for alternatives felt neonicotinoids “*have absolutely no place whatsoever in agriculture”*. The main perspective missing from this study due to non-response was from the sugar beet industry. Questions on respondents’ opinions resulted in identification of two main problems in this system, political polarisation and the monopsony of British Sugar.

RQ2 sought to determine how successful forecasting was in 2021 at reducing the use of neonicotinoids and maintaining crop success. This evaluation found that sugar beet crops had grown comparatively well in the absence of thiamethoxam, based on first-hand experience of growers, the BBRO’s review, and post-harvest yield statistics, suggesting that forecasting was accurate. Comparing this to the previous year (2020) when EU legislation continued the ban of thiamethoxam and high aphid numbers resulted in large volumes of authorised foliar insecticide spraying, yield was 26% higher in 2021 and in the absence of seed treatment and minimal spraying.

The study also identified limitations to the forecasting model. Growers were concerned that the forecast was published too late in the season, which may delay drilling. As it was the first year using the forecasts, it is also hard to know whether the threshold was set correctly, or the conditions were just favourable in 2021. The model is also determined at a national scale, which doesn’t accurately represent the complexity of pest population dynamics. Further research is being conducted at a spatial scale, however respondent 2 describes the process as ‘proving quite challenging’. This would also benefit from incorporating pollinator population dynamics, so that the model can work in parallel to local scale exposure predictions.

Considering what viable alternatives there are to the use of thiamethoxam seed treatment (RQ3), alternative pest management strategies were found to be limited in the *Myzus persicae* sugar beet system. IPM strategies such as biocontrols and crop rotations were considered too weak against the threat of virus yellows. It was even described by one respondent as working against the growers in this system, as encouragement of biocontrols tends to introduce new reservoirs for the virus.

HPR was also discussed, and described by one respondent as the ‘ideal solution’ based on it removing the need to control the pest at all. Ultimately however the genetic variation in sugar beet and virus yellows makes breeding for resistance extremely challenging. Having said that, a variety has recently been developed with partial tolerance to BMYV and will be available for use in 2022. This is an encouraging step forward, however partial tolerance is not sufficient, and so thiamethoxam is likely to still be considered.

A full ban was considered as another alternative, however it did not satisfy criteria regarding equity of stakeholder satisfaction and risk to growers. It also scored relatively low in minimising environmental impact, as banning the use of thiamethoxam effectively subjects the sugar beet crop to failure in years with high aphid forecasts, resulting in unintended environmental consequences such as foliar sprays and importation from countries with fewer regulations.

### Contribution of this research

This study is, to the best of our knowledge, one of the first to evaluate the use of virus yellows forecasting in informing pesticide regulatory decisions. It therefore provides a review for decision makers and illustrates a standard for alternative strategies to be accurately compared to, as was used here in comparative analysis. Identifying and synthesising the limitations of alternative pest management strategies in this system also provides a resource that may contribute to the public education and research considerations recommended.

More broadly, this study complicates the false dichotomy set up between food security in the form of productivist agriculture, and environmental protection, often observed across agricultural narratives (Chappell and LaValle, 2011). As in this case study, pesticide use is perceived to be distinct from sustainable agricultural practices, which is shown here to stunt progression toward a more agro-ecologically minded solution, through lack of communication and political polarisation (Lehrer and Sneegas, 2018). This study also highlights the importance of addressing the complexity of sustainable food production by opening up the discussion and taking a more nuanced and adaptive approach to policy.

The recommendations outlined here contribute to a comprehensive understanding of the UK sugar beet system, and urge the narrative to transition to a more nuanced, united, progressive one that can be applied and used as an example for future discussion on pesticides, food security and environmental protection.

## Data Availability

Participants were assured that interview transcript data remained confidential and not shared, so the data is not publicly available.

## Appendix

### Appendix 1. List of interviewees

**Table:**

Participant ID	Unspecified job title	Details of relevant research/work	Interview date	Interview duration
1	Insect molecular biologist	Research focusses on insecticide mode of action, resistance biochemistry, molecular basis of resistance and alternative control measures such as chemical ecology and IPM	08/07/2021	45 mins
2	Quantitative ecologist and entomologist	Research on forecasting the aphid vector of virus yellows, in collaboration with the BBRO.	05/07/2021	60 mins
3	Policy officer	UK charity organisation that is focused on tackling issues associated with pesticides. Work involves campaigning and trying to influence government decisions regarding pesticides	06/07/2021	40 mins
4	Consultant ecologist	Previously worked for a research organisation that ran field tests on pesticides, as well as research experience on the impacts of Thiamethoxam on bumblebees.	16/07/2021	60 mins
5	Independent agronomist	Advises farmers, including sugar beet growers, on pest management.	14/07/2021	40 mins
6	Entomologist	Specialises in developing new integrated pest management strategies in horticulture. Research has included finding alternatives to neonicotinoids.	20/07/2021	45 mins
7	Arable farmer	Grows sugar beet in South Lincolnshire. Uses neonicotinoid seed treatment when authorised.	22/07/2021	60 mins
8	Arable farmer	Grows sugar beet in South Lincolnshire. Uses neonicotinoid seed treatment when authorised	26/07/2021	40 mins
9	Arable farmer	Previously farmed sugar beet. Campaigns for sustainable British farming. Does not use neonicotinoids due to environmental impacts.	08/08/2021	90 mins

### Appendix 2. Interview Question Guide

#### SECTION 1: Introduction

- So, can you tell me about what you do and how you got into it?
- As you know my research is about neonicotinoids, what interactions do you have to Neonicotinoids in you professional life?
- What do you understand neonicotinoids to be?
  - Where did that understanding come from?
- What do you think about Neonicotinoids and how would you describe them?
- Where do you access information on Neonicotinoids?
  - Can you tell me why you use that source(s) primarily?
- How do you feel about the current decision to authorise emergency use of Thiamethoxam in England?
  - Why do you feel that way about it? What are your concerns?

#### SECTION 2: ‘The problem’

- The evidence for environmental impacts of Neonicotinoids is clearly quite convoluted and complex and seem to have divided stakeholder opinions. What is your perception of the environmental impacts?
- In what ways do you think that the current policies in England reflect the scientific evidence? And in what way?
- If they do not reflect scientific evidence, what else do you think those decisions were based on?
- Do you think that resistance to pesticides is a persistent issue? And do you think this is a concern for Neonicotinoids?
  - Can you tell me more about that?/ Please explain what you mean further
  - Why do you think that is?

#### SECTION 3: Alternatives to neonicotinoids

- What alternatives do you think there are to using Neonicotinoids?
  - How does that work?
  - Where is it from? Is it well researched/practiced/understood?
- Some of the literature suggests that IPM and neonicotinoids are mutually exclusive. Do you think this is the case? What do you think about it?
- Aphid forecasting is currently used to predict the extent of virus yellow in sugar beet, and in doing so limits the use of neonicotinoids when virulence is low. How effective do you think that this monitoring strategy is?
- With traditional IPM stages, monitoring is at the bottom and chemical pesticide use is a last resort. Why do you think that strategies in-between these such as biological controls or agronomic solutions have not been used more?
- Do you know anything about crop breeding for resistance to virus yellows in sugar beet? Is this a viable solution?
- What sort of weaknesses are there to plant breeding as a strategy for pest management?
- How do you think that farmers feel about pesticides? Of course they cannot all be grouped together but do you think they would prefer a more sustainable solution to pesticides?
  - Why is that?
  - What alternatives do you think would most preferable?
- What do you think are the best alternatives to pesticides, if any?
  - Why is that?
- What do you think would encourage farmers to adopt IPM strategies?

#### SECTION 4: Integrated Pest Management

- Do you think that IPM is used enough in the UK? Please explain.
  - Could it be used more?
  - What would they look like?
  - What are the barriers to it in the UK?
- Some say that IPM strategies are currently limited by a lack of encouragement and education to farmers. Would you agree with that?
  - Why is that?
  - What might help remedy this?

Close:

- I think those are all my questions
  Do you mind if I just take a moment to check that I have covered everything?
- Is there anything else you would like to add? Or think I have missed?
